# Dietary lactic acid and rosemary leaf supplementation enhances growth and immune responses in Nile tilapia (*Oreochromis niloticus*)

**DOI:** 10.1038/s41598-026-56341-8

**Published:** 2026-06-12

**Authors:** Essam Menshawy, Eman N. Abd Elazeez, Ibrahim M. Khattab, Safaa E. Abdo, Sabreen E. Fadl, Ghada A. El-Gammal, Manar B. Abd Elshafy, Mayada M. H. Khalil

**Affiliations:** 1 Department of Animal and Fish production, Faculty of Agriculture, Alexandria University, Alexandria, Egypt; 2 Department of Animal and Fish Production, Faculty of Desert and Environmental Agriculture, Matrouh University, Matrouh, Egypt; 3 Genetics and Genetic Engineering, Department of Animal Wealth Development, Faculty of Veterinary Medicine, Kafrelsheikh University, Kafrelsheikh, Egypt; 4 Biochemistry Department, Faculty of Veterinary Medicine, Matrouh University, Matrouh, Egypt; 5 Bacteriology Unit, Kafrelsheikh Regional Laboratory, Animal Health Research Institute, Agricultural Research Center (ARC), Giza, Egypt

**Keywords:** Nile tilapia, Lactic acid, Rosemary leaf, Growth performance, Antioxidant status, Immunity, Biochemistry, Physiology, Zoology

## Abstract

The present study investigated the individual and combined effects of lactic acid and rosemary meal on growth performance, biochemical parameters, immune responses, the expression of growth and antioxidant-related genes, intestinal morphology, and oxidative status in Nile tilapia (*Oreochromis niloticus*). A total of 120 apparently healthy fish, with an average weight of 3.03 ± 0.02 g, were randomly assigned to four equal groups, each consisting of three replicates. Four experimental diets were formulated: a basal control diet (CON), a basal diet supplemented with 1 g of lactic acid per kg of diet (LA), a basal diet containing 10 g of rosemary per kilogram of diet (RM), and a basal diet that included both supplements (LA + RM). Fish were fed these diets for a duration of 60 days. The results indicated that either lactic acid or rosemary alone or in combination had a greater growth-stimulating impact than the CON group (*P* ≤ 0.05) with superiority to the combination group (LA + RM group). Activities of aspartate aminotransferase and alanine aminotransferase, along with creatinine and urea levels, were significantly reduced (*P* ≤ 0.05) in the groups of lactic acid and rosemary alone or in combination relative to the CON group. Total protein and albumin concentrations were elevated in the LA + RM group (*P* ≤ 0.05). Intestinal histology revealed normal morphology across groups, with increased villus height, intestinal villi spacing, and goblet cell density in LA + RM (*P* ≤ 0.05) without pathological lesions in the liver and spleen. Antioxidant, immune, and growth-related gene expressions were upregulated in RM and LA + RM groups. In conclusion, rosemary supplementation, alone or combined with lactic acid, enhanced fish health status and upregulated target genes without pathological lesions with superiority to the combination treatment.

**Supplementary Information:**

The online version contains supplementary material available at 10.1038/s41598-026-56341-8.

## **Introduction**

Aquaculture production is experiencing rapid growth worldwide, leading to an increased use of antibiotics and other chemicals that can be harmful to both consumers and the environment. Aquaculture productivity is thus confronted by environmental stressors and diseases, necessitating effective feed additives to bolster fish health and performance^1^, (Abdulaziz et al., 2024). Natural compounds, such as herbal essential oils, probiotics, and medicinal herbs, have gained significant attention as sustainable alternatives to synthetic additives. These natural options have shown benefits for growth, immunity, and overall health in aquatic organisms^2–4^. Additionally, in aquaculture, medicinal plants are being studied as a safe and environmentally friendly way to improve growth performance, avoid fish disease, and modify immunological state^5^. Grape seed extract and cornelian cherry supplementation have the potential to greatly improve common carp growth performance, immunological responses, and disease resistance against *Aeromonas hydrophila*^6^and^7^; respectively). However, the ability of plant extracts to improve fish health and disease resistance makes them environmentally benign alternatives to traditional treatments in aquaculture^8,9^.

Specifically, rosemary leaf has emerged as a promising phytogenic additive due to its antioxidant and immunostimulatory properties, which are mainly attributed to its rich carnosic and rosmarinic acid content^10^. These bioactive compounds contribute to enhanced growth performance, improved haematological indices, and better immune responses in various fish species, including Nile tilapia^11,12^. Several studies support the effectiveness of such phytogenic additives, showing increased growth rates, improved feed conversion ratios, and enhanced disease resistance in tilapia aquaculture^13,14^. The immunomodulatory effects of medicinal plants, such as rosemary, stem from secondary metabolites like phenolic, polyphenolic, and alkaloid compounds, which can stimulate both innate and adaptive immune responses (Mohammed et al.,, 2024). While lowering the use of chemicals and antibiotics in aquatic ecosystems, natural polyphenols can be regarded as reasonably safe and practical substitutes for synthetic chemical compounds that not only improve fish health status but also improve fish quality, productivity, and food safety^15^.

Similarly, lactic acid, a common organic acid, has gained recognition for its ability to modulate gut microbiota, enhance nutrient absorption, and contribute to overall host health. This indicates a synergistic potential when combined with herbal extracts^16^. Similar synergistic effects have been observed in other aquatic species, where the combination of plant-based extracts and organic acids has optimized physiological functions and increased disease resistance^17,18^.

This comprehensive approach, which combines phytogenics and organic acids, offers a compelling strategy for sustainable aquaculture by reducing stress and improving biological parameters^19^. Therefore, this study aimed to investigate the individual and combined effects of lactic acid and rosemary meal on growth performance, biochemical parameters, immune responses, the expression of growth and antioxidant-related genes, intestinal morphology, and oxidative status in Nile tilapia.

## Materials and methods

This study was approved by Faculty of Agriculture, Alexandria University, Egypt (Serial No. 0108540). We obtained permission through an email from the owner to use the fish in our study.

### Fish husbandry conditions, diet preparation, and experimental design

A total of 120 apparently healthy Nile tilapia (*Oreochromis niloticus*) were procured from a local farm and acclimatized for two weeks in 300-L fiberglass tanks at the research facility of the Department of Animal and Fish Production, Faculty of Agriculture, Alexandria University. Following acclimation, fish were randomly selected for the experiment.

Fish were allocated to four treatments across 12 glass aquaria, with 10 individuals per aquarium and three replicates per treatment. The experimental treatments included the control group (CON) fed a basal diet without additives; the LA group supplemented with lactic acid (85% purity; molecular weight, 90.08 g/mol, Sigma-Aldrich, USA) at 1 g/kg diet according to El-Dakar et al.^20^ the RM group supplemented with rosemary leaf powder at 10 g/kg diet according to Mohammed et al.^21^ and the LA + RM group supplemented with both additives. Rosemary (*Rosmarinus officinalis L*.) leaves were procured from a local market in Aswan Governorate, Egypt. The fresh foliage was thoroughly washed with distilled water and subsequently air-dried in a shaded, well-ventilated area. The material was inverted periodically until a constant weight was achieved. The fully dehydrated leaves were then pulverized using a Wiley mill to obtain a uniform powder (Table S1). All dietary ingredients were thoroughly blended to formulate the experimental diets, which were then pelleted using a laboratory pelletizer. Post-preparation, the diets were dried at ambient temperature and stored at 4 °C. The ingredients and composition of the basal experimental diet are shown in Table 1.

**Table 1:** Ingredients and chemical composition of the basal diet.

Item	Content, %
Ingredients	
Soybean meal	20
Yellow corn	20
Fish meal (68%)	17
Rice bran	15
Wheat bran	10
Corn gluten	10
Plant oil	6
Vitamin and Minerals mixture	2
Chemical composition	
Crude protein	34.4
Crude lipid	9.81
Ash	17.2
Crude fiber	2.22
Nitrogen free extract	36.3

Fish were hand-fed twice daily (9:00 a.m. and 1:00 p.m.) at 3% of their body weight throughout a 60-day experimental period. The aquarium water was partially renewed daily with dechlorinated freshwater, maintaining a temperature of approximately 24 °C. Dissolved oxygen, salinity, and pH were maintained at 5.8–6.1 mg/L, 1.1–2‰, and 7.4–8.1, respectively.

### Growth indices and feed utilization

Fish were weighed at the beginning of the experiment and then every two weeks for a total of 60 days. The growth parameters were calculated using the following equations:

- Weight gain (G) = Final body weight - Initial body weight.
- Average daily gain (ADG) = (W1 - W0)/T, where T represents the number of days in the experimental period, while W1 denotes the final body weight in grams according to Castell and Tiews^22^.
- Feed Intake (g/fish): The amount of feed offered during the experimental period/fish (g).
- Feed conversion ratio (FCR) = Feed intake (g)/Weight gain (g)^23^.

### Chemical analysis

Five fish were collected from each replicate or group to assess the chemical composition of their bodies. The experimental diet and fish samples were weighed and then dried at 65 °C for 24 h. The samples were analysed for dry matter (Method 934.01), ash (Method 942.05), crude protein (Method 954.01), ether extract (Method 920.39), and crude fiber in diet (Method 978.10) according to the Society of Official Analytical Chemists AOAC^24^. Nitrogen-free extract of the diet was calculated by subtracting the percentages of ash, crude fibre, ether extract, and crude protein from 100.

###  Biochemical blood indices

At the end of the experimental trial, five fish from each replicate were randomly selected, and blood was drawn from the caudal vein. The blood sample was centrifuged at 3000 rpm for 10 min, and the serum was kept at − 20 °C in storage. Serum parameters were measured colorimetrically for aspartate aminotransferase (AST: CAT. No. AS 1061) and alanine aminotransferase (ALT: CAT. No. AL 1031) according to Reitman and Frankel^25^ total protein (CAT. NO. TP 20 20), and albumin (CAT. No. AB 10 10) according to Young^26^ and globulin was calculated by subtracting albumin from total protein. Additionally, urea and creatinine, according to Reitman and Frankel^27^ total antioxidant capacity (TAC: CAT. No. TA 2513), and malondialdehyde (MDA: CAT. No. MD 2529) levels were assessed according to Koracevic et al.^28^ and Ohkawa et al.^29^ respectively, along with superoxide dismutase (SOD: CAT. No. SD 2521) and catalase (CAT: CAT. No. CA 2517) activities according to Nishikimi et al.^30^ and Aebi^31^ respectively, using commercial colorimetric kits from Bio-Diagnostics Company in Egypt, following the manufacturer’s guidelines.

In addition to intestine, liver, and spleen histology, this assessment was conducted by assessing liver and kidney function.

### Histopathology and intestinal morphometry

At the end of the experimental trial, five fish per replicate were randomly selected and humanely euthanized via immersion in an overdose bath of clove oil. Upon complete cessation of opercular movement, the liver, spleen, and intestine were carefully excised. 10% neutral buffered formalin was used to fix the samples. The tissues were then dehydrated, cleared, and then fixed in paraffin before being sectioned into 5-µm-thick slices. Hematoxylin and eosin staining was applied to these serial sections^32^.

The histomorphometry analysis was performed using ImageJ analysis software (National Institutes of Health, MD, USA). Measurements of the intestinal villi length, width, and inter-villi space were performed by ImageJ analysis software and expressed in µm. The program is available for free online at https://imagej.nih.gov/ij/download.html.

### Gene expression

After being collected in liquid nitrogen, liver samples were stored at −80 °C until analysis. The RNA was first extracted using Trizol (iNtRON Biotechnology, Inc., Korea) and then inspected through 2% ethidium bromide-stained agarose gel electrophoresis and Nanodrop spectrophotometry (260 nm wavelength). The cDNA was synthesized complementary to the mRNA using a reverse transcription-specific kit (2X RT Mix, Applied Biotechnology, Egypt).

The mRNA levels were evaluated using the quantitative real-time PCR (PikoReal, Thermoscientific, TCR0024) and the 2x -Lo-Rox- SYBR Green kits (Applied Biotechnology, Egypt). A set of genes specific primer pairs was used for various gene categories, including some growth-related genes (*GHR1* and *IGF1*), antioxidant genes (*CAT* and *GPX*), and innate immune response genes (*LZM* and *C3*). *Ef-1α* served as the internal control gene. Primer sequences and accession numbers are listed in Table 2. According to Abdo et al.^36^, the reaction mix preparation and thermocycler conditions were carried out. The relative expression was calculated as a fold change compared to the control group (non-supplemented), according to the method outlined by Livak and Schmittgen^37^.

**Table 2:** The primers used in the gene expression.

Gene	Primer	Slope	Efficiency %	Product size	Accession NO	Reference
Elongation factor-1α (*ef-1α*)	F: TCAACGCTCAGGTCATCATC R: ACGGTCGATCTTCTCAACCA	−3.32	100.08	125 bp	XM_003458541	Abdo et al^20^.
Catalase *(cat)*	F: CCCAGCTCTTCATCCAGAAAC R: GCCTCCGCATTGTACTTCTT	−3.41	96.45	103 bp	JF801726.1	El-Kassas et al^33^.
Glutathione peroxidase *(gpx)*	F: CCAAGAGAACTGCAAGAACGA R: CAGGACACGTCATTCCTACAC	−3.39	97.24	237 bp	DQ355022	
Lysozyme gene *(lzm)*	F: AAGGGAAGCAGCAGCAGTTGTG R: CGTCCATGCCGTTAGCCTTGAG	−3.31	100.50	151 bp	XM_003460550.2	Dawood et al^34^.
Complement 3 (*c3)*	F: GGTGTGGATGCACCTGAGAA R: GGGAAATCGGTACTTGGCCT	−3.369	9 98.07	163 bp	XM_013274267.2	
*Growth hormone receptors 1(ghr1)*	F: CAGACTTCTACGCTCAGGTC R: CTGGATTCTGAGTTGCTGTC	−3.35	98.84	80 bp	MW509678.1	Hamed et al^35^.
Insulin-like growth factor 1 (*igf1)*	F: GTTTGTCTGTGGAGAGCGAGG R: GAAGCAGCACTCGTCCACG	−3.35	99.25	97 bp	NM_001279503.1	

*Ef-1α*: elongation factor-1α (*ef-1α*); *CAT*: Catalase; *GPX*: glutathione peroxidase; *GHR1*: growth hormone receptors 1; *IGF-1*: insulin-like growth factor 1; *LZM*: lysozyme gene; *C3*: complement 3.

### Statistical analysis

Normality and homoscedasticity were demonstrated using the Shapiro-Wilk and Levene tests. After that, one-way ANOVA was used to examine the effects of additives on *O. niloticus*. The differences among means were tested using the Tukey test as a post hoc test. Average values ± standard deviation (SD) were used to express the experimental results. All analyses were performed using the SPSS software statistical package (SPSS version 26.0 for Windows). The least significant difference was calculated at *P* ≤ 0.05^38^.

## Results

### Growth performance

Table 3 shows the effects of supplementing the feed of *O. niloticus* with lactic acid and rosemary on BW at various time points. At 2 and 4 weeks, the BW in the LA + RM group showed a significant (*P* ≤ 0.05) increase compared with the CON and LA and LA and RM groups, respectively. Meanwhile, at 6 weeks, LA + RM treatment resulted in a notable increase in BW (*P* < 0.05) compared with the CON, LA, and RM groups. However, the final BW in both RM and LA + RM treatments was higher than that of the CON and LA groups. Other growth performance metrics, including weight gain, feed intake, ADG, and FCR, showed improvements in the RM and LA + RM groups; however, these changes were not statistically significant. On the other hand, the survival rate significantly (*P* ≤ 0.05) increased in the LA and LA + RM groups when compared with the CON and RM groups.

**Table 3:** The effect of feed additives on the growth performance of Nile tilapia (*Oreochromis niloticus*) throughout the experimental period.

Parameters	Groups			
	CON	LA	RM	LA + RM
Initial body weight (g)	3.03 ± 0.03	3.02 ± 0.05	3.05 ± 0.03	3.01 ± 0.08
Body weight 2 weeks	4.77 ± 0.18^b^	4.75 ± 0.09^b^	4.90 ± 0.06^ab^	5.25 ± 0.14^a^
Body weight 4 weeks	7.48 ± 0.31^ab^	6.98 ± 0.25^b^	7.17 ± 0.18^b^	8.14 ± 0.12^a^
Body weight 6 weeks	9.50 ± 0.49^b^	8.68 ± 0.25^b^	9.42 ± 0.09^b^	10.93 ± 0.10^a^
Final body weight (g)	11.03 ± 0.87^b^	11.08 ± 0.45^b^	10.93 ± 0.10^a^	13.86 ± 0.37^a^
Weight gain (g)	8.00 ± 0.52	8.06 ± 0.19	8.56 ± 0.01	10.85 ± 0.40
Feed intake (g)	17.57 ± 0.63	16.72 ± 0.40	17.34 ± 0.19	19.35 ± 0.01
Feed conversion ratio	2.21 ± 0.10	2.07 ± 0.01	2.03 ± 0.02	1.79 ± 0.07
Average daily gain (g/day)	0.133 ± 0.009	0.134 ± 0.003	0.143 ± 0.001	0.181 ± 0.007
Survival rate	96.67 ± 3.33 ^b^	100 ± 0.00 ^a^	96.67 ± 3.33 ^b^	100 ± 0.00 ^a^

Values are means ± standard error. Mean values with different letters at the same row significantly differ at (*P* ≤ 0.05). CON group: the basal diet; LA group: the basal diet with 1% lactic acid; RM group: the basal diet with 10 g of rosemary/kg of diet; LA + RM group: the basal diet with both lactic acid and rosemary.

### Body composition

Body composition across groups, including crude protein, crude fat, and ash, was not significantly affected by dietary treatments. However, moisture levels were significantly (*P* ≤ 0.05) higher in the LA + RM group compared to the LA group (Table 4).

**Table 4:** Effect of feed additives on whole body composition (dry-weight basis) of Nile tilapia fed experimental diets for 60 days.

Parameters	Groups			
	CON	LA	RM	LA + RM
Moisture	73.43 ± 0.39^ab^	72.64 ± 0.34^b^	73.42 ± 0.27^ab^	74.22 ± 0.51^a^
Crude protein	57.76 ± 0.67	57.16 ± 0.11	58.26 ± 0.28	57.99 ± 0.12
Crude lipid	19.87 ± 0.25	19.56 ± 0.33	19.48 ± 0.69	19.65 ± 0.36
Ash	22.36 ± 0.26	23.28 ± 0.22	22.26 ± 0.68	22.35 ± 0.48

Values are means ± standard error. Mean values with different letters at the same row significantly differ at (*P* ≤ 0.05). CON group: the basal diet; LA group: the basal diet with 1% lactic acid; RM group: the basal diet with 10 g of rosemary/kg of diet; LA + RM group: the basal diet with both lactic acid and rosemary.

### Serum biochemical indices

Activities of AST and ALT were significantly (*P* ≤ 0.05) reduced in the LA, RM, and LA + RM groups relative to the CON group, with the LA + RM intervention demonstrating the most substantial decrease. In *O. niloticus*, total protein and albumin concentrations were elevated in all treatment groups compared to the CON group, wherein the LA + RM treatment exhibited the highest values. Conversely, globulin levels were elevated by dietary LA and RM relative to both the CON and LA + RM groups. Urea concentrations were markedly lower in the LA, RM, and LA + RM groups than in the CON group, while creatinine levels were significantly (*P* ≤ 0.05) decreased in the RM and LA + RM groups compared to the CON (Table 5).

**Table 5:** Effect of feed additives on serum biochemical parameters of Nile tilapia fed experimental diets for 60 days.

Parameters	Groups			
	CON	LA	RM	LA + RM
AST (U/L)	63.5 ± 0.87^a^	55±0.1.15 ^b^	54.5 ± 0.29 ^b^	46.5 ± 0.87 ^c^
ALT (U/L)	27.5 ± 1.44 ^a^	23 ± 0.58 ^b^	22 ± 1.15 ^b^	18.5 ± 0.29 ^c^
Total protein (g/dl)	3.80 ± 0.026 ^c^	3.99 ± 0.02 ^b^	4.01 ± 0.04 ^b^	4.38 ± 0.02 ^a^
Albumin (g/dl)	1.27 ± 0.01 ^b^	1.32 ± 0.02 ^b^	1.32 ± 0.003 ^b^	1.9 ± 0.02 ^a^
Globulin (g/dl)	2.53 ± 0.01 ^b^	2.67 ± 0.003 ^a^	2.70 ± 0.04 ^a^	2.48 ± 0.01 ^b^
Creatinine (mg/dl)	1.03 ± 0.01 ^a^	0.97 ± 0.009 ^ab^	0.89 ± 0.61 ^b^	0.65 ± 0.03 ^c^
Urea (mg/dl)	5.14 ± 0.07 ^a^	4.5 ± 0.20 ^b^	4.61 ± 0.14 ^b^	3.21 ± 0.03 ^c^

Values are means ± standard error. Mean values with different letters at the same row significantly differ at (*P* ≤ 0.05). CON group: the basal diet; LA group: the basal diet with 1% lactic acid; RM group: the basal diet with 10 g of rosemary/kg of diet; LA + RM group: the basal diet with both lactic acid and rosemary.

### Antioxidative status

The MDA levels in the CON, LA, and RM groups were higher (*P* ≤ 0.05) than in the LA + RM group. Moreover, the LA + RM group showed significant (*P* ≤ 0.05) interactive effects in CAT and TAC. Furthermore, CAT and TAC significantly (*P* ≤ 0.05) increased in the LA and RM groups compared with the CON group (Table 6).

**Table 6:** Effect of feed additives on serum antioxidant and oxidative stress of Nile tilapia fed experimental diets for 60 days.

Parameters	Groups			
	CON	LA	RM	LA + RM
MDA (nmol/ml)	4.71 ± 0.32^a^	4.50 ± 0.08 ^a^	4.6 ± 0.01 ^a^	3.09 ± 0.02 ^b^
CAT (U/ml)	2.38 ± 0.13 ^c^	2.8 ± 0.03 ^b^	2.87 ± 0.05 ^b^	5.30 ± 0.02 ^a^
TAC (ng/ml)	0.82 ± 0.04 ^c^	1.01 ± 0.03 ^b^	0.93 ± 0.01 ^b^	1.48 ± 0.03 ^a^

Values are mean ±stander error. Mean values with different letters at the same row significantly differ at (*P* ≤ 0.05). CON group: the basal diet; LA group: the basal diet with 1% lactic acid; RM group: the basal diet with 10 g of rosemary/kg of diet; LA + RM group: the basal diet with both lactic acid and rosemary.

### Histopathology

**Fig. 1:**
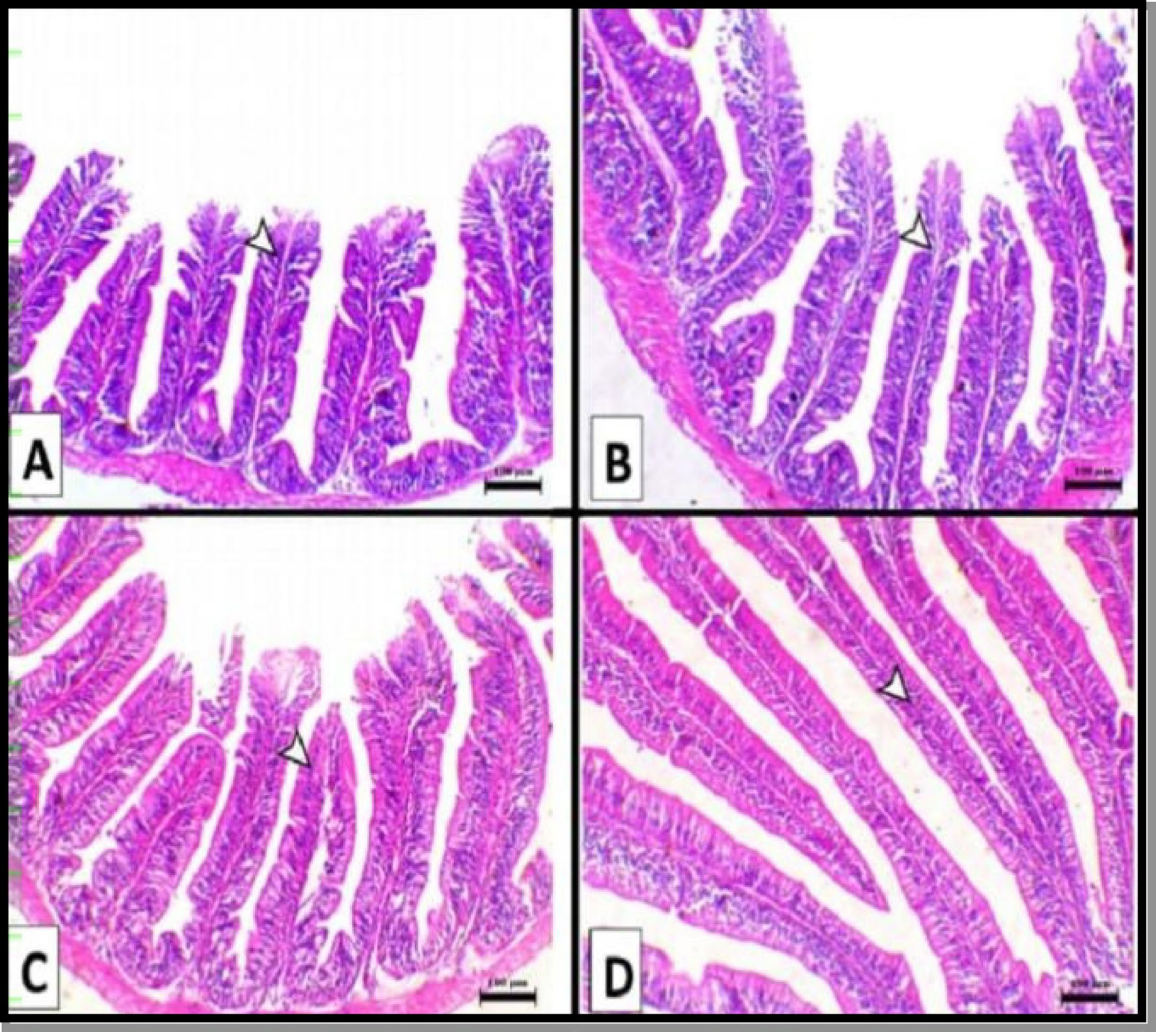
Shows the middle portion of the fish intestine of the different treatments. (**A**) Intestine of the control group showing normal villi lined with pseudostratified epithelium with goblet cells (arrowhead). (**B**) and (**C**) Intestine of the LA and RM groups, respectively, showing mild increase of intestinal villi length (arrowhead). (**D**) Intestine of the LA + RM group showing marked increase of intestinal villi length (arrowhead), H&E, X100, bar = 100 μm.

Figure 1 shows the middle portion of the fish intestine of the different treatments. Intestine (middle portion) of the CON group showing normal villi lined with pseudostratified epithelium with goblet cells (A). The LA and RM groups showed a mild increase (*P* ≤ 0.05) in intestinal villi length (B and C, respectively). The LA + RM group showed a marked increase in intestinal villi length (D). However, Table 7 shows significant (*P* ≤ 0.05) interactive effects of dietary LA, RM, and LA + RM on villi length and goblet cells of Nile tilapia, with priority to the LA + RM treatment. Meanwhile, inter-villus space significantly (*P* ≤ 0.05) decreased in the LA + RM group compared with the other groups. Moreover, the villi width showed no significant differences between treatments.

**Table 7:** Effect of feed additives on intestinal morphometry of Nile tilapia fish fed experimental diets for 60 days.

Parameters	Groups			
	CON	LA	RM	LA + RM
Villi length (um)	386.79 ± 0.60 ^d^	458.82 ± 0.74 ^c^	471.36 ± 3.93 ^b^	751.60 ± 0.32^a^
Villi width (um)	82.47 ± 3.35	87.56 ± 3.23	75.50 ± 4.28	76.66 ± 4.82
Inter villi space (um)	64.47 ± 1.85 ^b^	61.88 ± 0.71 ^b^	62.84 ± 3.24 ^b^	47.75 ± 1.42 ^a^
Goblet cells (No/mm^2^)	142 ± 3.22 ^d^	170 ± 2, 89 ^c^	202.33 ± 4.67 ^b^	398 ± 1.73 ^a^

Values are mean ±stander error. Mean values with different letters at the same row significantly differ at (*P* ≤ 0.05). CON group: the basal diet; LA group: the basal diet with 1% lactic acid; RM group: the basal diet with 10 g of rosemary/kg of diet; LA + RM group: the basal diet with both lactic acid and rosemary.

**Fig. 2:**
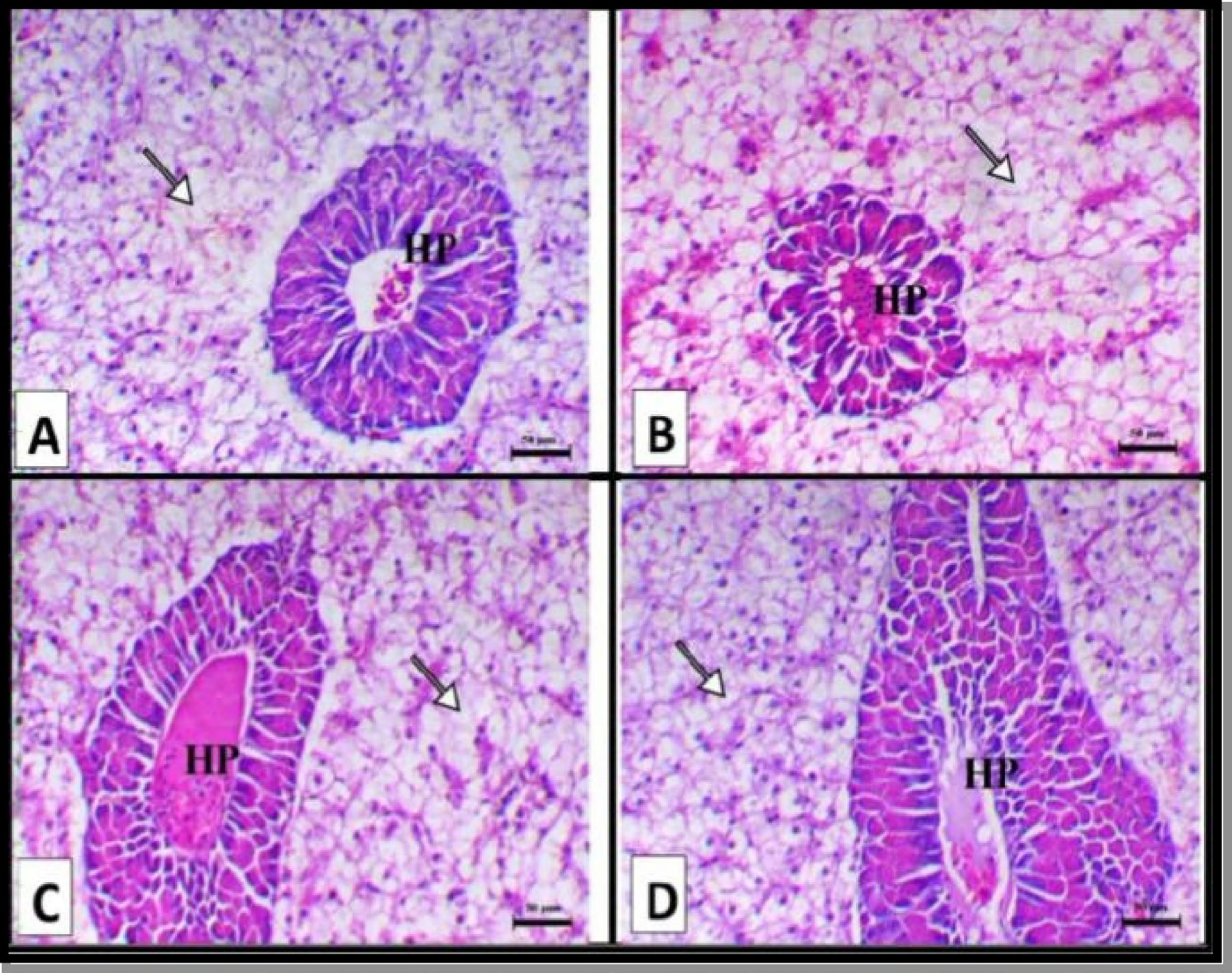
Shows liver tissue of the different treatments. (**A**) and (**B**) Liver of fish of control and LA groups, respectively, showing normal hepatic and pancreatic portions (HP) with marked hepatic vacuolation (arrow). (**C**) Liver of fish of the RM group showing normal hepatic and pancreatic portions (HP) (arrow indicates marked hepatic vacuolation). (**D**) Liver of fish of the LA + RM group showing normal hepatic and pancreatic portions (HP) (arrow indicates decrease hepatic vacuolation), H&E, bar = 50 μm.

Figure 2 shows liver tissue for the different treatments. The liver of fish of the CON and LA groups shows normal hepatic and pancreatic portions with a marked hepatic vacuolation (A and B, respectively). Meanwhile, livers of fish of the RM and LA + RM groups showed normal hepatic and pancreatic portions (C and D, respectively).

**Fig. 3:**
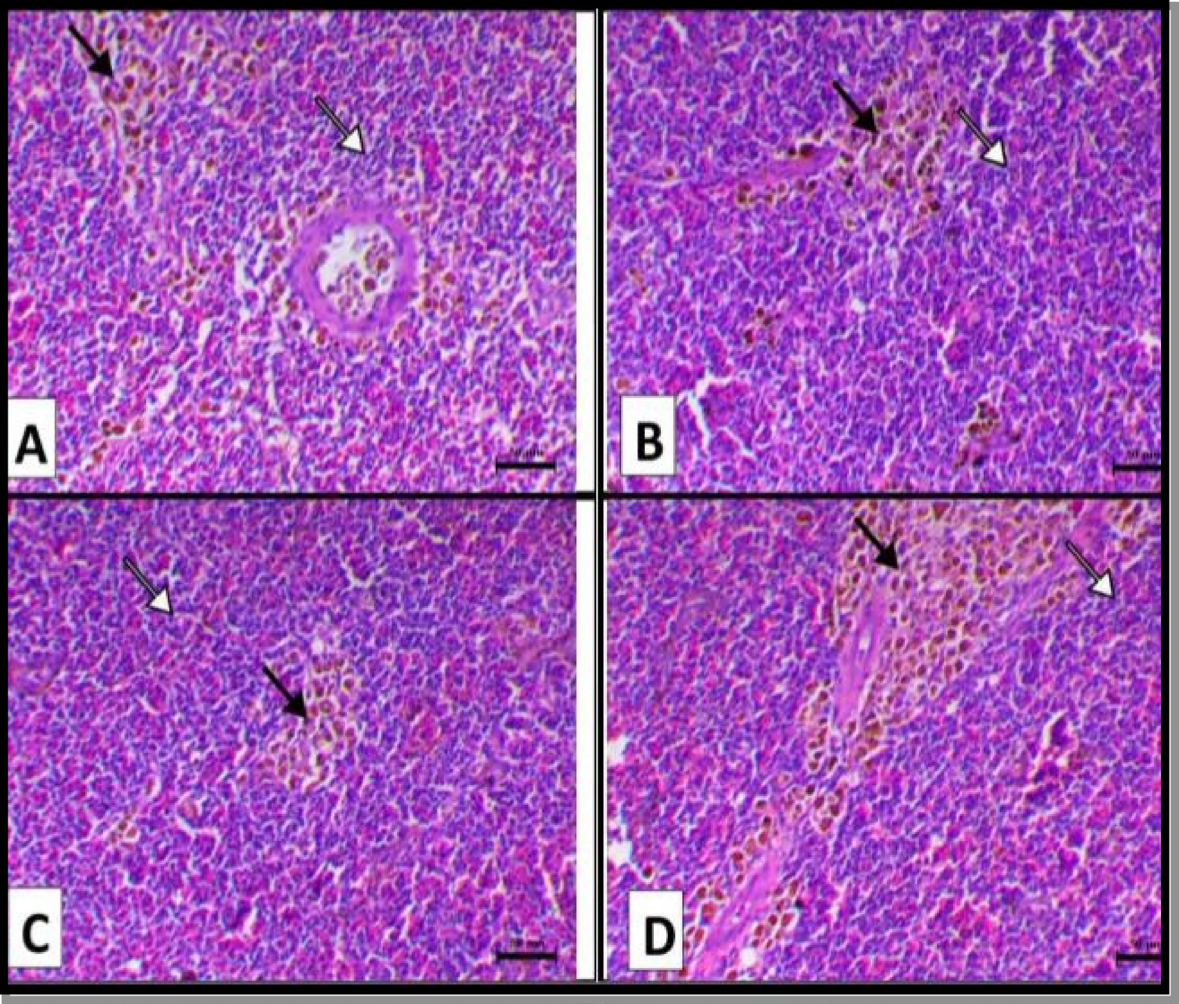
Shows spleen tissue of the different treatments. (**A**) and (**B**): Spleen of fish of control and LA groups, respectively, showing normal lymphoid tissues including normal melanomacrophages cells (arrowhead) (arrow). (**C**) Spleen of fish of the RM group showing normal lymphoid tissues including normal tiny foci of melanomacrophages cells (arrowhead) (arrow). (**D**) Spleen of fish of the LA + RM group showing normal lymphoid tissues (white arrow) with marked increase of melanomacrophages cells (black arrow), H&E, bar = 50 μm.

Figure 3 shows spleen tissue for the different treatments. Spleens of fish of the CON and LA groups showed normal lymphoid tissues, including normal melanomacrophage cells (A and B, respectively). The spleen of fish of the RM group showed normal lymphoid tissues, including normal tiny foci of melanomacrophage cells (C). The spleens of fish of the LA + RM group showed normal lymphoid tissues with a marked increase of melanomacrophage cells (D).

### Gene expression

**Fig. 4:**
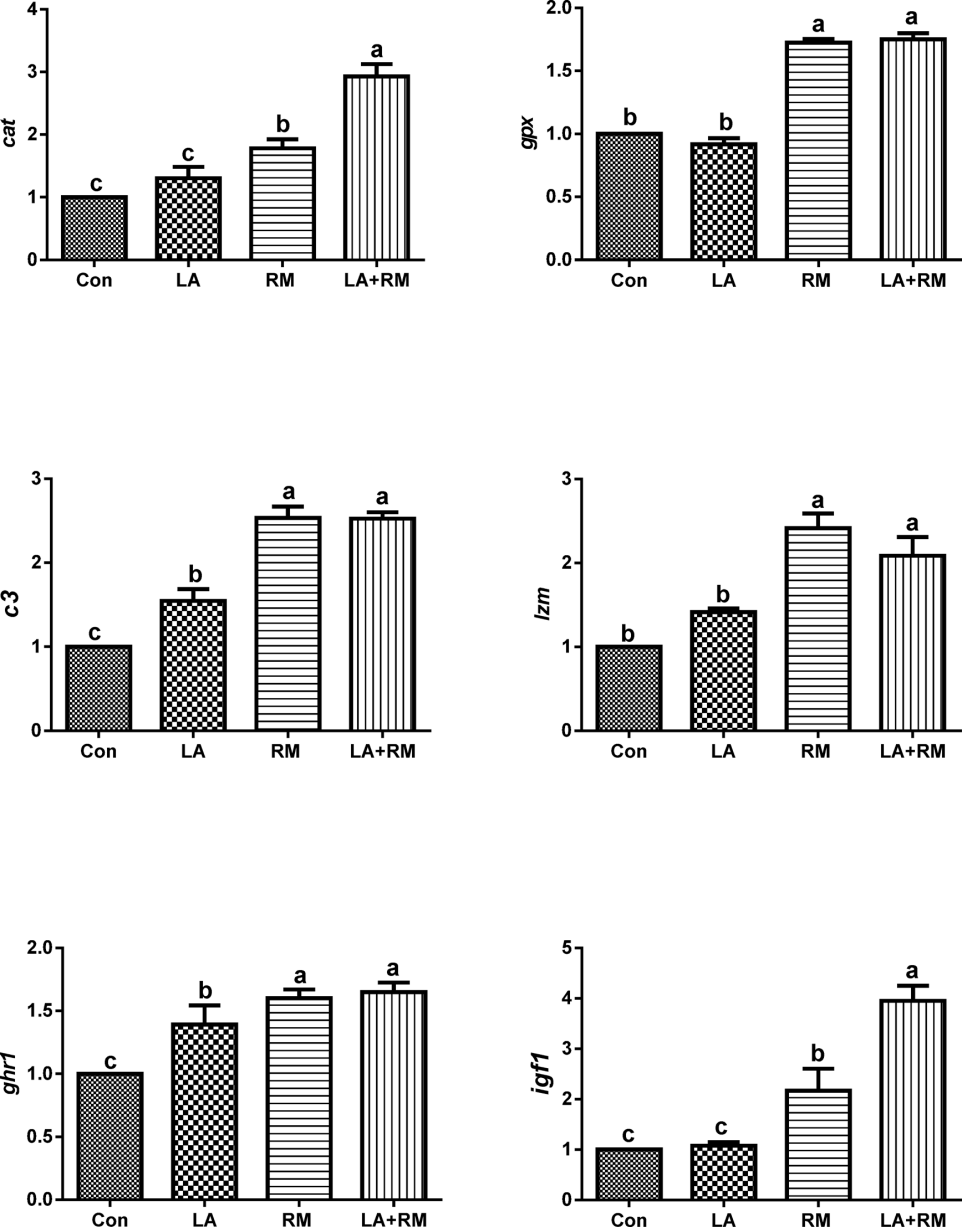
Showed the expression of the antioxidant-related genes (*CAT*,* GPx*), innate immunity-related genes (*C3* and *LZM*), and growth-related genes (*GHR1*,* IGF1*) in the various treatments.

The expression of the antioxidant-related genes (*CAT* and *GPx*) is depicted in Fig. 4, where the RM and LA + RM groups exhibit overexpression of *CAT* and *GPx*. Conversely, the *LZM* was upregulated in the RM and LA + RM groups, as demonstrated by the innate immunity-related genes *C3* and *LZM*. In the meantime, the LA, RM, and LA + RM groups showed upregulation of *C3*. In the RM and LA + RM groups, there was an overexpression of the growth-related genes (*GHR1* and *IGF1*). In the meantime, the LA, RM, and LA + RM groups showed upregulation of *GHR1*.

## Discussion

Nowadays, one of the main sources of rational animal protein required for human consumption is aquaculture, which is in higher demand^39^. In addition, there have been suggestions to reduce or eliminate the use of chemical stimulants like hormones and antibiotics as feed additives in fish diets in favour of herbs and herbal supplements, which are more cost-effective, environmentally benign, and have fewer negative effects^40^. Additionally, fish feeds containing organic acids provide a number of advantages, including reduced feed microbial load, improved nutritional digestibility, intestinal development, dominance of beneficial microorganisms in the fish intestine, and strengthened immune systems^41^. The results of the LA on the growth contrast with the findings of Hoseini et al.^42^ who found that LA (5–20 g/kg diet) in the diet of the rainbow trout does not affect growth performance. Medium-chain fatty acids (MCFAs) did not significantly affect white shrimp growth^43^. The effects of dietary LA on the growth of Nile tilapia were not comparable to those observed in common carp, which exhibited a change in growth performance when provided dietary LA at a rate of 5 g/kg diet^44^. Abd Elshafy^1^ and Sobhy et al. and^45^ reported that medium-chain fatty acids and LA, respectively, significantly improved the growth performance of the Nile tilapia. These variations could result from species-specific characteristics, age of fish, dose of the organic acids, duration of use, and nutritional makeup. However, the same beneficial impact of the rosemary on the BW^12,21,46^. Rosemary leaf powder dietary supplementation improved the growth of common carp^47^.‏ Moreover, Turan and Yiğitarslan^48^ reported the promoting effect of rosemary extract on the BW of the African catfish. This positive effect may be attributed to the beneficial impact of the rosemary on the digestive system. (Huang^49^,) reported the use of rosemary to improve nutrient digestion and utilization efficiency. However, these results were confirmed by the results of the intestinal morphometry. Moreover, rosemary contains flavonoid and phenolic acid molecules that can improve intestinal shape, block deamination and dehydrogenation processes during bacterial growth in vivo, and boost the body’s capacity to utilize nutrients^50^. Moreover, rosemary stimulates liver function and pancreatic secretion^51^. On the other hand, the non-significant effect of rosemary on the other growth performance is incompatible with Hassan et al.^14^ who observed the significant effect of rosemary on the weight gain. However, as far as we are aware, no studies have been conducted on the application of LA and rosemary in combination for fish production. (Abdo^52^,) noted that the Nile tilapias fed a combination of lemongrass and spirulina had the maximum growth performance, indicating that their effects were synergistic. Additionally, the growth-promoting effect of a combined therapy with rosemary and spirulina in Nile tilapia was documented by Mohammed et al.^21^. Ahmadifar^53^ found that goldfish development, immunity, and disease resistance are enhanced by nutritional supplementation with 0.5 g/Kg chlorogenic acid and 10^10^ CFU/g *Saccharomyces boulardii* separately and in combination. However, findings regarding the gene expression of the growth-related genes support the growth performance results.

Nutritional status has an impact on the overall body composition of fish. The findings of the body composition in the LA group are similar to the findings of Mirghaed et al.^44^ who reported that LA does not significantly affect the body composition of the common carp. Also, Zhang et al.^54^, found that organic acid has not significantly affected whole-body composition in American eel or red sea bream, respectively. On the other hand, Omar^47^ reported that dietary rosemary increased moisture and did not affect the crude fat and ash of the common carp chemical composition but decreased crude protein. According to Hassan et al.^14^ when Nile tilapia was fed a diet containing rosemary extracts, their moisture contents significantly differed from those of the control group. On the other hand, when Nile tilapia was given a meal containing rosemary extracts, Yilmaz et al.^10^ found no discernible variation in the amount of fat and ash levels when compared to the control fish fed. Additionally, rosemary powder did not significantly change common carp’s protein and fat content^55^.

The present finding comes with the finding of Magouz et al.^56^ and Abd Elshafy et al., and^2^, who observed that MCFA reduced AST and ALT activities in Nile tilapia and common carp, respectively. Additionally, Zhang et al.^57^ used medium-chain triglycerides in pigs and found similar results. Agouz et al.^58^ observed that organic acid supplementation in *O. niloticus*did not affect liver enzyme activity and increased serum protein. Additionally, Abd Elshafy et al.^2^ found that MCFA increased serum protein in Nile tilapia with normal kidney function. Hussein^59^ reported that organic acids in the diet of *Sparus aurata* did not affect serum liver enzymes, creatinine, and urea.‏ On the other hand, the findings of the liver enzyme activities of the RO group are compatible with the findings of Omar^47^ who found that *O. niloticus*and common carp fed rosemary had lower liver enzyme activities, respectively. After 12 weeks of therapy, Hernandez et al.^60^ found that *Sparus aurata* supplemented with rosemary extract had lower ALT activities. Treatments’ hepatoprotective effects could be the cause of these outcomes. Total protein and albumin levels were increased in a dose-dependent manner in fish fed a diet containing rosemary extracts^61^.

Because of the high levels of unsaturated fatty acids in the fish bodies, oxidative stress is one of the deadliest conditions that can affect fish^62^. The finding of the antioxidant and oxidative stress of the LA group is consistent with the results of Chen et al.^63^ who demonstrated that dietary LA supplementation dramatically raises the activity of antioxidant enzymes and lowers fish MDA levels. Hoseini et al.^64^ observed that dietary LA improves antioxidant and intestinal health. Additionally, studies have indicated that beneficial bacteria can increase fish’s antioxidant capacity, which may be linked to the antioxidant-modulating impact of dietary LA supplementation and an improvement in the fish gut microbiota^65,66^. On the other hand, Naiel et al.^67^ reported that dietary rosemary leaf powder enhances antioxidant properties in *O. niloticus*. Moreover, antioxidant enzyme activities were markedly elevated in a dose-dependent way when rosemary was provided^68^. However, the antioxidant was pronounced in the combination group.

The liver histology results validated the findings of the serum liver function tests. This study also discovered a strong correlation between AST and ALT activity and liver microscopy results. Similar results are obtained by Han et al.^69^ and Ullah et al. and^70^ in weaned piglets and *black sea bream*, respectively, with MCFA, who revealed that, without causing any pathological alterations in the intestine, the administration of MCFA improved several aspects of intestinal histoarchitecture, such as villus height and goblet cell numbers. Additionally, Zhang et al.^57^found that medium-chain triglycerides did not affect the liver histology of pigs. On the other hand, Khalil et al.^68^ observed that intestinal morphometry is improved by rosemary extract. Findings regarding the gene expression of the antioxidant-related genes, however, support the antioxidant results.

(Meng^71^,) observed that, as a component of the innate immune system, complement has long been understood to protect the host from the invasion of foreign infections. *C3*(complement component 3) is a key element in the complement system. These are confirmed by the antioxidant and immune-related gene expressions in the present investigation. Karataş et al.^72^, found that rainbow trout’s immunology, antioxidant enzymes, and general health are all improved by rosemary. Reduced levels of antioxidant and immunological indices are suggestive of DNA and cellular damage brought on by the buildup of ROS, which causes oxidative stress and immune suppression^11^. El-Kassas^34^ found in healthy fish that supplementing the diet with powdered *Moringa oleifera* leaves can increase the immune system and decrease the inflammatory response linked to infection. Moreover, in the therapy groups, the innate immunity-related genes were elevated. Similar outcomes were observed by Ebrahimi et al. (2020) using essential oil of rosemary in *Huso huso*. Zargar^73^ found that supplementing with essential oils from *Thymus vulgaris* increased the transcription of genes related to the immune system. Additionally, common carp fed *M. oleifera*had significantly stronger innate immune response features, according to Adeshina et al.^74^. The bioactive compounds found in rosemary may be responsible for these outcomes^75^. *gcC3 mRNA* expression levels were markedly elevated in the liver of grass carp following *Aeromonas hydrophila* infection^71^. This indicates that this gene has increased resistance to infection. On the other hand, Fontinha et al.^35^, reported the positive impact of short-chain fatty acids on the immune and antioxidant-related genes in *Dicentrarchus labrax*. However, this positive effect was pronounced in the combination group (LA + RM). A similar finding was reported by Mohammed et al.^21^ in Nile tilapia with combination treatment by spirulina and rosemary. Additionally, giving *O. niloticus* a rosemary and cinnamon mixture (10 g/kg diet) protect them from oxidative damage, physiological changes, and stress brought on by lead nitrate^11^. On the other hand, Abdo et al.^52^ indicated that Nile tilapias fed lemongrass and spirulina had the strongest immunological response, indicating that their activities were synergistic.

Fish growth is mostly controlled by growth hormone (*GH*) and insulin-like growth factor I (*IGF-I*)^76^. Numerous factors, including an organism’s environment, genetic makeup, and diet, affect these genes^77^. Despite the small number of studies, it has been shown that the addition of phyto plants stimulates the expression of growth genes like *GH* and *IGF*^78^. Furthermore, it has been demonstrated that phytochemicals boost immune-related gene expression, which in turn stimulates the animal’s immune system. Fish grow more quickly as a result of these advantageous effects of phytochemicals, which also improve health and disease resistance^78,79^. However, El-Kassas et al.^33^ observed that performance was enhanced by the proportionate rise in growth hormone receptor and IGF mRNA levels that occurred after the addition of moringa leaves to the meal. Also, Abd Elshafy et al.^1^ observed the positive impact of the short-chain fatty acids on the growth-related genes of *O. niloticus*. Xuan^80^ reported that for *O. niloticus* raised in a biofloc system, adding 10 g/kg of powdered avocado seed to the feed significantly improves growth, immunological function, and gene expression. Compared to non-fed common carp, those fed malic acid showed higher expression of genes linked to growth^81^. Additionally, Abdel-Tawwab et al.^82^ found that juvenile Nile tilapia fed sodium butyrate nanoparticles produced comparable results. However, this positive effect was pronounced in the combination group (LA + RM). These results align with the findings of Choi et al.^83^ a substantial increase in *Juvenile olive flounder’s*growth-related gene upon the addition of four feed supplements. Moreover, Abd Elshafy et al.^1^, observed the positive impact of the combination of the three feed additives on the growth-related genes of *O. niloticus*. However, rosemary supplementation in Nile tilapia has been shown to positively affect gene expression, particularly by upregulating immune and antioxidant-related genes like *IL-1*,* IL-8*,* LBP*,* GST-α*,* GPX*, and *GSR*^80^. Lactic acid’s specific effect on Nile tilapia gene expression is less directly documented, though it is often studied in combination with other supplements and can positively influence growth, immunity, and antioxidant defence, as seen with some probiotic strains like *Lactobacillus plantarum*^84^.

## Conclusion

All things considered, the dietary supplements of rosemary and combination treatments employed in this experiment were found to have a wide range of positive benefits. Growth performance, chemical composition, and the expression of genes linked to antioxidants, immunity, and growth were all positively impacted by rosemary, either separately or in combination, with normal liver and splenic architecture. Additionally, intestinal villi and goblet cells significantly increased in the combination group. The favourable effects of the combination treatment on the growth indices of final BW, WG, ADG, FI, and FCR supported this result. The *CAT*,* GPx*,* C3*,* LZM*,* GHR1*, and *IGF1* genes are also upregulated. Each of the two modifications (lactic acid and rosemary) showed promising results on its own, providing a potentially useful way to boost aquaculture growth.

## Supplementary Information

Below is the link to the electronic supplementary material.

Supplementary Material 1

## Data Availability

The datasets used and/or analyzed during the current study available from the corresponding author on reasonable request.

## Declarations

### AI statement

During the revision of this work the authors used Grammar Checker in order to improve the language and used AI for the preparation of graphical abstract. After using this tool, the authors reviewed and edited the content as needed and takes full responsibility for the content of the published article.
