# Supplementary material for: Dietary lactic acid and rosemary leaf supplementation enhances growth and immune responses in Nile tilapia (Oreochromis niloticus)
Source: Sci Rep. 2026 Jun 12;16:18327. doi: 10.1038/s41598-026-56341-8 (PMC13263333; doi:10.1038/s41598-026-56341-8)
Supplement: Supplementary file 1 — Supplementary Material 1 [file 41598_2026_56341_MOESM1_ESM.docx]

**Table S1: List of the active components of rosemary leaf powder**

| Phenolic acids | Rosmarinic acid, Caffeic acid, Chlorogenic acid, protocatechuic acid, 4-hydroxybenzoic acid, and methylrosmarinate | Fakhri et al., 2022  Zhang and Lu, 2024. |
| --- | --- | --- |
| Terpenoids | Monoterpenes comprise linear types such as geraniol and nerol, monocyclic varieties including thymol, carvacrol, and limonene, and bicyclic forms such as 1,8-cineole, α-pinene, and camphor.  Sesquiterpenes.  Diterpenes are further categorized into diterpenoid phenols–including carnosic acid, carnosol, and rosmanol.  Pentacyclic triterpenes, delineated by aglycone structure into oleanane-type (e.g., oleanolic acid), ursane-type (e.g., ursolic acid), and lupane-type (e.g., betulinic acid). Oleanolic acid and ursolic acid, as structural isomers, differ in methyl group positioning (C-20 and C-19, respectively). | Miljanović et al., 2023  Mlala et al., 2019 Žiberna et al., 2017 |
| Flavonoids | There are more than 30 flavonoid compounds. These include flavones (e.g., apigenin, genkwanin, and luteolin), flavonols (e.g., quercetin, isorhamnetin, and kaempferol), dihydroflavones (e.g., hesperetin, hesperidin, and naringin), and dihydroflavonols (e.g., gallocatechin). | Liu et al., 2024  Naimi et al., 2017  Sok et al., 2021 |
| Other active components | Rosemary extracts contain various organic acids and fatty hydrocarbons, such as vanillic acid and gallic acid. Amino acids as asparagine, threonine, alanine, tyrosine, phenylalanine, isoleucine, and proline and essential minerals such as potassium (K), sodium (Na), iron (Fe), calcium (Ca), magnesium (Mg), and trace elements including phosphorus (P) and sulfur (S). | Liu et al. (2025) |

**References**

Fakhri S., Abbaszadeh F., Moradi S.Z., Cao H., Khan H., Xiao J. Effects of polyphenols on oxidative stress, inflammation, and interconnected pathways during spinal cord injury. Oxid Med Cell Longev. 2022;2022

Liu C., Huang H., Chen Y., Zhou Y., Meng T., Tan B., He W., Fu X., Xiao D. Dietary supplementation with mulberry leaf flavonoids and carnosic acid complex enhances the growth performance and antioxidant capacity via regulating the p38 MAPK/Nrf2 pathway. Front Nutr. 2024;11

Liu, Z., Xia, T., Jiang, A., Zhou, C., Lukuyu, B. A., & Tan, Z. (2025). Biological functions and applications of rosemary extracts in animal production. Animal Nutrition.‏

Miljanović A., Dent M., Grbin D., Pedisić S., Zorić Z., Marijanović Z., Jerković I., Bielen A. Sage, rosemary, and bay laurel hydrodistillation by-products as a source of bioactive compounds. Plants. 2023;12:2394. doi: 10.3390/plants12132394.

Mlala S., Oyedeji A.O., Gondwe M., Oyedeji O.O. Ursolic acid and its derivatives as bioactive agents. Molecules. 2019;24:2751. doi: 10.3390/molecules24152751.

Naimi M., Vlavcheski F., Shamshoum H., Tsiani E. Rosemary extract as a potential anti-hyperglycemic agent: current evidence and future perspectives. Nutrients. 2017;9:968. doi: 10.3390/nu9090968.

Sok Yen, F., Shu Qin, C., Tan Shi Xuan, S., Jia Ying, P., Yi Le, H., Darmarajan, T., ... & Salvamani, S. (2021). Hypoglycemic effects of plant flavonoids: a review. Evidence‐Based Complementary and Alternative Medicine, 2021(1), 2057333.‏

Zhang L., Lu J. Rosemary (Rosmarinus officinalis L.) polyphenols and inflammatory bowel diseases: major phytochemicals, functional properties, and health effects. Fitoterapia. 2024;177(Sep)

Žiberna L., Šamec D., Mocan A., Nabavi S.F., Bishayee A., Farooqi A.A., Sureda A., Nabavi S.M. Oleanolic acid alters multiple cell signaling pathways: implication in cancer prevention and therapy. Int J Mol Sci. 2017;18:643. doi: 10.3390/ijms18030643.
